# Nanoparticle-based vaccine development and evaluation against viral infections in pigs

**DOI:** 10.1186/s13567-019-0712-5

**Published:** 2019-11-06

**Authors:** Santosh Dhakal, Gourapura J. Renukaradhya

**Affiliations:** 1 Food Animal Health Research Program, Ohio Agricultural Research and Development Center, 1680 Madison Avenue, Wooster, OH 44691 USA; 2 Department of Veterinary Preventive Medicine, College of Veterinary Medicine, The Ohio State University, Columbus, OH 43210 USA

## Abstract

Virus infections possess persistent health challenges in swine industry leading to severe economic losses worldwide. The economic burden caused by virus infections such as Porcine Reproductive and Respiratory Syndrome Virus, Swine influenza virus, Porcine Epidemic Diarrhea Virus, Porcine Circovirus 2, Foot and Mouth Disease Virus and many others are associated with severe morbidity, mortality, loss of production, trade restrictions and investments in control and prevention practices. Pigs can also have a role in zoonotic transmission of some viral infections to humans. Inactivated and modified-live virus vaccines are available against porcine viral infections with variable efficacy under field conditions. Thus, improvements over existing vaccines are necessary to: (1) Increase the breadth of protection against evolving viral strains and subtypes; (2) Control of emerging and re-emerging viruses; (3) Eradicate viruses localized in different geographic areas; and (4) Differentiate infected from vaccinated animals to improve disease control programs. Nanoparticles (NPs) generated from virus-like particles, biodegradable and biocompatible polymers and liposomes offer many advantages as vaccine delivery platform due to their unique physicochemical properties. NPs help in efficient antigen internalization and processing by antigen presenting cells and activate them to elicit innate and adaptive immunity. Some of the NPs-based vaccines could be delivered through both parenteral and mucosal routes to trigger efficient mucosal and systemic immune responses and could be used to target specific immune cells such as mucosal microfold (M) cells and dendritic cells (DCs). In conclusion, NPs-based vaccines can serve as novel candidate vaccines against several porcine viral infections with the potential to enhance the broader protective efficacy under field conditions. This review highlights the recent developments in NPs-based vaccines against porcine viral pathogens and how the NPs-based vaccine delivery system induces innate and adaptive immune responses resulting in varied level of protective efficacy.

## Economically important viral infections of pigs

Viruses are the obligate intracellular nano-sized particles, which depend on host cell machinery for propagation and survival. They carry deoxyribonucleic acid (DNA) or ribonucleic acid (RNA) as their genomic material. There are several viruses from both DNA and RNA virus families that infect and produce disease in pigs [1]. There are many economically important swine viral infections which cause considerable morbidity and mortality, and responsible for significant economic losses to the pork industry (Table 1). Depending on their cellular and tissue tropisms, viruses cause pathological changes and clinical signs associated with respiratory system, reproductive and gastrointestinal tracts, nervous system, skin and extremities, alone or in combinations [1, 2].

**Table 1:** **Economically important viral diseases of pigs**

Disease	Virus	Epidemiology	Clinical signs	References
Porcine reproductive and respiratory syndrome (PRRS)	PRRS virus (PRRSV) Single strand (+) RNA	Worldwide	Fever, anorexia, mild to severe respiratory problems, abortion, reproductive failures	[3]
Swine influenza	Influenza A virus (IAV) Single strand (−) RNA	Worldwide	Fever, anorexia, loss of weight gain, respiratory problems	[5, 28]
Porcine epidemic diarrhea (PED)	PED virus (PEDV) Single strand (+) RNA	Worldwide	Severe diarrhea, vomiting and dehydration	[115]
Foot and mouth disease (FMD)	FMD virus (FMDV) Single strand (+) RNA	Parts of Asia, Africa, Middle East and South America	Fever, inappetence, vesicular lesions on extremities	[11]
Classical swine fever (CSF)/hog cholera	CSF virus (CSFV) Single strand (+) RNA	Endemic in Central America, Africa, Asia and parts of South America	Fever, anorexia, erythema, respiratory signs, neurological signs, reproductive failures, death	[13]
Porcine circovirus associated disease (PCVAD)	Porcine circovirus 2 (PCV2) Single strand DNA	Worldwide	Poor weight gain, respiratory problems, dermatitis, enteritis, nephropathy, reproductive failures	[16]
Porcine parvovirus infection	*Ungulate parvovirus 1* Single strand DNA	Worldwide	Stillbirth, mummification, embryonic death, infertility	[17]
Pseudorabies/Aujeszky’s disease	*Suid herpesvirus 1* Double strand DNA	China and parts of Europe, Asia and Latin America	Nervous disorders, respiratory problems, weight loss	[18, 19]
African swine fever (ASF)	ASF virus (ASFV) Double strand DNA	Endemic in sub-Saharan Africa, Sardinia, Caucasus region and Eastern Europe	Fever, anorexia, erythema, respiratory signs, reproductive failures, death	[13]

### Diseases caused by RNA viruses

Porcine reproductive and respiratory syndrome virus (PRRSV), an enveloped and positive-stranded RNA virus of *Arteriviridae* family, causes porcine reproductive and respiratory syndrome (PRRS) [3]. PRRS is responsible for over one billion dollar loss per year through direct and indirect costs in the US swine industry [4]. Two entirely distinct genotypes of PRRSV circulate in European (genotype 1/PRRSV 1) and North American countries (genotype 2/PRRSV 2) and cause tremendous economic loss. PRRSV is transmitted through oral-nasal secretions and semen. The clinical signs include fever, anorexia, mild to severe respiratory problems, abortion and reproductive failures. It is the most common pathogen associated with porcine respiratory disease complex (PRDC) [3].

Swine influenza (flu) constitutes another persistent health challenge to the global pig industry. Flu infection is caused by influenza A virus of Orthomyxoviridae family which has negative-sense, single-stranded, segmented RNA genome. Influenza virus is transmitted through direct contact with infected animals or contaminated fomites, aerosols and large droplets [5]. The clinical signs of influenza infection include fever, anorexia, loss of weight gain and respiratory problems. Influenza associated economic losses are due to morbidity, loss of body weight gain, increased time to market, secondary infections, medication and veterinary expenses [6]. Influenza of swine origin occasionally infect humans and can even lead to pandemics as of 2009 [7].

Porcine epidemic diarrhea virus (PEDV), transmissible gastroenteritis virus (TGEV) and porcine deltacoronavirus (PDCoV) are enteric pathogens of young pigs [8]. These viruses belong to *Coronaviridae* family and have positive-sense, single-stranded RNA genome. TGEV did serious economic damage to the swine industry in 1990s but with the advent of vaccines it has been largely controlled [8]. PEDV still results in high morbidity and mortality in neonatal piglets with clinical signs like severe diarrhea, vomiting, dehydration and death. In 2013/14, PEDV outbreak in the US led to over a billion-dollar loss [9]. Rotaviruses are double-stranded RNA viruses of *Reoviridae* family, cause enteric infections in pigs. Rotavirus of groups A, B, C, E and H are involved in porcine enteric infections. Some of these porcine rotaviruses also have zoonotic potential [10].

Foot and mouth disease (FMD) is another highly contagious, acute viral disease in pigs. The etiologic agent, FMD virus (FMDV), is a positive-sense, single-stranded RNA virus of *Picornaviridae* family [11]. FMDV is transmitted through direct contact with infected animals or contaminated sources. Clinical signs include high fever, appearance of vesicular lesions on the extremities, salivation, lameness and death. FMDV causes frequent epizootics in many parts of the world resulting in severe economic loss, food insecurity and trade restrictions [11].

Classical swine fever (CSF) or hog cholera can result in high morbidity and mortality in pigs. It is caused by CSF virus (CSFV), an enveloped, positive-sense, single-stranded virus of *Flaviviridae* family. Transmission of CSFV occurs through oral-nasal routes after contact with infected pigs or contaminated resources and even vertically from infected sows to piglets [12]. Clinical signs include fever, anorexia, respiratory problems, neurological disorders, reproductive failures and death. CSF is a notifiable disease to World Organization for Animal Health (OIE). The economic losses are associated with production loss, trade limitations and tremendous expenditures in eradication programs [13]. For example, the 1997/98 outbreak of CSFV in the Netherland resulted in death of 9 million pigs and economic losses of 2.3 billion dollars [14]. United States is free of CSFV; however, this virus is endemic in many parts of the world including Central and South America, Africa and Asia.

### Diseases caused by DNA viruses

Porcine circovirus 2 (PCV2), a single-stranded DNA virus of *Circoviridae* family, causes multi-systemic disease referred as porcine circovirus-associated disease (PCVAD). PCV2 is transmitted horizontally as well as vertically. Direct contact is the most efficient way of horizontal transmission of this virus. The clinical signs of PCV2 infection include poor weight gain, respiratory problems, dermatitis, enteritis, nephropathy and reproductive failures [15]. Five genotypes of PCV2 (PCV2a to PCV2e) are identified and circulate with high prevalence in swine herds causing significant economic losses worldwide [16].

Porcine parvovirus (PPV) is the common cause of reproductive failure in swine herds. This single-stranded DNA virus of *Parvoviridae* family is transmitted through oral-nasal routes. Stillbirths, mummification, embryonic death, and infertility (SMEDI syndrome) are linked to PPV infection. Conventionally, PPV was considered genetically conserved but recent evidences suggest that several virulent strains have emerged due to its high mutation rate [17].

Aujeszky’s disease or pseudorabies in pigs is caused by Suid herpesvirus 1, a double stranded DNA virus belonging to *Herpesviridae* family. The causative agent is spread primarily through direct animal-to-animal (nose-to-nose or sexual) contact. Pseudorabies is characterized by nervous disorders, respiratory problems, weight loss, deaths in younger piglets and reproductive failures; and is one of the most devastating infectious diseases in pig industry [18, 19].

African Swine Fever (ASF) causes hemorrhagic infection with high morbidity and mortality. The etiologic agent, ASF virus (ASFV), is a double stranded DNA virus of *Asfarviridae* family [20]. Virus transmission occurs through direct contact with infected animals, indirect contacts with fomites or through soft tick species of the genus *Ornithodoros*. Clinical disease may range from asymptomatic infection to death with no signs. Acute infections are characterized by high fever, anorexia, erythema, respiratory distress, reproductive failure in pregnant females and death [20]. ASF is OIE notifiable disease. United States is free of ASFV, however, this virus is endemic in domestic and wild pig population in many parts of the world with possibility of transmission to the US and other nonendemic regions through animal trades [13]. The economic losses are associated with production loss, trade limitations and tremendous expenditures in eradication programs [13].

Besides the RNA and DNA viruses described above, many other emerging and re-emerging viruses such as porcine hepatitis E virus, porcine endogenous retrovirus, porcine sapovirus, Japanese encephalitis virus, encephalomyocarditis virus and others cause variable degree of impact in swine health and economic losses in pig industry globally [2, 21, 22].

## Vaccination against porcine viral infections

Different types of vaccines that are available against economically important swine viruses are listed in Table 2. Vaccines against PRRSV are being used in the US since 1980s [23]. Both inactivated and modified-live virus vaccines are available and used globally. These vaccines are effective in reducing clinical disease and viremia mainly against homologous but not against heterologous infections [24]. Therefore, different strategies are ongoing to develop live, inactivated, subunit and mucosal PRRSV vaccines to induce better immunity and broader protection [23, 25–27]. Swine influenza vaccines are also most effective when the vaccine strains closely match with the circulating strains [5, 28].

**Table 2:** **Vaccines available against economically important porcine viral infections**

Disease	Vaccines available	Improvements needed	References
Porcine reproductive and respiratory syndrome (PRRS)	Inactivated, modified-live virus	Rapid immune induction Heterologous protection No adverse impact on health	[23, 24]
Swine influenza	Inactivated, modified-live virus	Broader protection No maternal antibody interference No vaccine-enhanced disease	[5, 28]
Porcine epidemic diarrhea (PED)	RNA particle, inactivated and live-attenuated virus (in Asia)	Protective immune response in sows Better mucosal immunity	[8, 9]
Foot and mouth disease (FMD)	Inactivated virus	Less stringent requirements in vaccine production Protection against multiple serotypes	[29]
Classical swine fever (CSF)	Live-attenuated virus	DIVA potential	[30]
Porcine circovirus associated disease (PCVAD)	Inactivated, recombinant subunit	Multi-genotype protection	[16, 31]
Porcine parvovirus infection	Inactivated virus	Protection against novel strains	[17, 33]
Pseudorabies	Inactivated, live-attenuated virus	Protection against novel emerging strains	[18, 19]
African swine fever (ASF)	None	Novel cross-protective vaccine	[20]

To increase the immunity and protection, vaccines containing multiple strains of influenza A virus (IAV) and autogenous vaccines are widely used [5, 28]. Co-circulation of multiple lineages of IAV and frequent antigenic drift are responsible for reduced field efficacy of current swine influenza vaccines. Moreover, the most commonly used whole inactivated IAV vaccines administered via intramuscular route do not induce adequate mucosal antibody and cellular immune responses, suffer maternal antibody interference in young piglets and even can cause enhanced respiratory disease [5, 28].

The emergence of highly virulent strains of PEDV in recent years has highlighted the need of safe and effective vaccines against porcine enteric coronaviruses that prevents clinical disease, mortality and virus shedding in neonates [8]. Modified live vaccines against rotavirus are available for use in pigs against rotavirus A but their efficacy under field conditions is questionable indicating the need of alternatives for porcine rotavirus management [10].

The available inactivated vaccines provided great help in prevention and control of FMD outbreaks in many countries. However, the development of these vaccines needs high level biocontainment facilities. Further, the FMDV serotypes undergo continuous antigenic drift and escape the vaccine-induced immunity [29]. Thus, FMD vaccines with less stringent regulatory procedures and multi-serotype protective efficacy are needed in the future. Safe and highly efficacious live-attenuated vaccines are available against CSFV but differentiation of infected from vaccinated animals (DIVA) is not possible with these vaccines, which limit their use during outbreak control or disease eradication programs [30].

Inactivated whole virus or subunit vaccines based on PCV2a are highly adopted in pig farms and are efficacious in reducing clinical signs and improving the production parameters. However, infections are still widespread in vaccinated farms [16, 31]. Further, the replacement of PCV2a to PCV2b and recently to PCV2d is in part contributed by the selection pressure exhibited by PCV2a-based vaccines [32] which highlights the need of vaccines that protect against multiple genotypes.

The currently used inactivated vaccines of porcine parvo virus protect against old PPV strains but not against the newly emerging strains demanding for more efficacious vaccines [17, 33]. Fortunately, pseudorabies has been eradicated in many countries including the US by using inactivated and attenuated vaccines together with stringent biosecurity measures. However, it is still a problematic disease in many countries including China and is also maintained in feral swine populations in other countries [18, 34]. The frequent emergence of virulent strains even in the vaccinated herds demands updated vaccine technology to achieve efficient control and ultimate global eradication of pseudorabies [19, 34].

Vaccine is not available so far against ASFV, and the control measures depend entirely on early identification and culling of infected herds and adoption of strict sanitary measures [35]. Vaccine development is hindered by the antigenic diversity and multitude of immune-evasion strategies used by the virus. An effective vaccine will definitely help in control and eradication of ASFV from endemic countries and prevent its geographical expansion [20].

## Importance of nanoparticle-based vaccine delivery platforms

**Figure 1:**
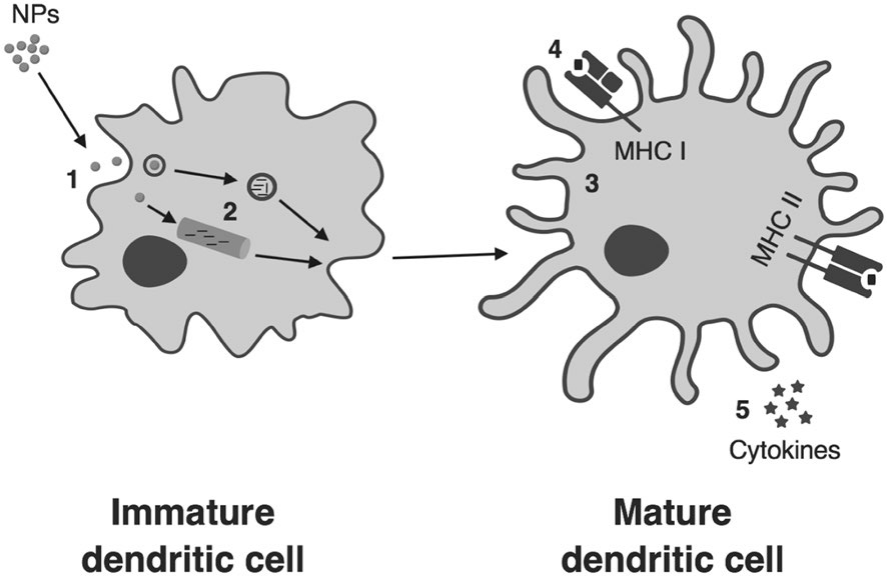
**Schematic representation of different effects imparted by NPs on APCs.** NPs-based vaccines can—(1) enhance antigen uptake; (2) facilitate antigen processing; (3) induce maturation of DCs; (4) promote antigen cross-presentation by MHC-I; and (5) induce cytokine production.

Development of vaccines has made significant impact on reducing the viral infectious disease burden in both humans and animals. However, there are still many diseases for which either we do not have vaccines or need substantial improvements over existing ones [36, 37]. In the past few decades, nanoparticles (NPs)-based technologies have elicited significant interests in the development of novel vaccine candidates as they offer multiple benefits over inactivated virus or subunit soluble antigens. NPs-based vaccines (nanovaccines) are prepared either by encapsulating vaccine components within the NPs or by decorating the particle surface with viral antigens. NPs protect antigens from proteolytic degradation, prolong their bioavailability and maintain slow and sustained antigen release. All of these properties help in induction of better immune responses compared to soluble antigen vaccines [38]. The different mechanisms used by various NPs to facilitate immune modulation of antigen presenting cells (APCs) are depicted graphically in Figure 1. Briefly, NPs can enhance antigen adsorption and uptake by APCs; they can also facilitate antigen processing mechanisms; NPs can induce maturation of DCs and promote antigen cross-presentation through major histocompatibility complex (MHC) class I to CD8^+^ T cells; and induce production of different innate cytokines that regulate humoral and cellular immune responses. NPs-loaded antigens are readily phagocytosed by APCs; soluble antigens are not [39]. Moreover, dendritic cells (DCs), the key player involved in bridging innate and adaptive immunity, preferentially internalize NPs compared to microparticles (> 1000 nm). For example, when poly(lactic-co-glycolic acid) (PLGA) particles of size 300 nm to 17 µm encapsulating ovalbumin were tested on mouse bone-marrow derived dendritic cells, 300 nm sized particles were taken up efficiently compared to larger ones [40]. The 300 nm sized PLGA NPs resulted in greater activation of DCs and stronger antigen-specific T cells responses in immunized mice compared to soluble antigens and larger particles [40].

Besides controlled delivery of antigens, NPs also provide adjuvant-like functions. Vaccine adjuvants either work as antigen delivery systems facilitating antigen uptake and presentation by APCs or they activate innate immune receptors for cytokine production and maturation/migration of DCs [41]. Adjuvant-induced innate immune responses determine the type of adaptive immune responses generated such as T helper 1 (Th1) versus T helper 2 (Th2)-biased immunity [42]. Alum, the most widely used adjuvant in humans, is safe and inexpensive. Its compatibility has been proved favorable with different vaccine antigens. However, despite inducing potent antibody responses, alum is a weak-inducer of cell-mediated immunity. Adverse reactions are observed at injection site with alum-based adjuvants [43, 44]. In veterinary vaccines, oil-in-water emulsions or saponins are the most common adjuvants. These can also cause adverse reactions at the injection sites [45, 46]. While number of adjuvants are available for parenteral vaccinations, very limited options are available for intranasal (IN) or other alternative routes of immunization [41, 47, 48]. NPs can serve as an alternative adjuvant for human and animal use as they act both as antigen delivery system and activate the innate immune responses [49–51]. Further, the modern vaccination approach has shifted from traditional whole pathogen-based antigens to small fraction (subunit) of the pathogen. However, purified whole inactivated pathogen and subunit or recombinant antigens by themselves are poorly immunogenic and require a potent immunostimulatory platform to augment the immune response. This can be achieved through NPs-based technologies [47, 52].

NPs-based platforms can be used to deliver multiple antigens or antigen/adjuvant combinations, which improves antigen uptake and concurrent activation of APCs leading to innate immune programming [53, 54]. Co-delivery of CpG oligodeoxynucleotide and tetanus toxoid in nanospheres induced significantly greater T cell proliferative response and 5 to 16 times greater IgG antibody isotypes in mice after subcutaneous immunization compared with the group that received tetanus toxoid and CpG oligodeoxynucleotide in soluble form [54]. Likewise, co-delivery of melanoma antigen and Toll-like receptor (TLR) 4 agonist in PLGA NPs induced therapeutic anti-tumor effects that are mediated through potent CD8^+^ T cell activation [55]. NPs can be surface modified to target microfold (M) cells, macrophages or DCs, and could be used for mucosal vaccination through oral, nasal or other mucosal routes of immunization. In mice, surface coating of PLGA NPs encapsulating hepatitis B virus vaccine antigens with lectin resulted in efficient targeting of oral delivered NPs to mucosal M cells and induced secretary IgA antibody response in mucosal surfaces [56]. Likewise, DCs targeted chitosan NPs loading plasmid DNA encoding nucleocapsid protein of Severe Acute Respiratory Syndrome Coronavirus (SARS-CoV) induced better nucleocapsid protein-specific mucosal IgA antibody response compared to soluble unentrapped antigens after nasal immunization in mice [57].

A targeted T-cell mediated immune response is critical in protection against intracellular pathogens such as viruses. Beneficially, NPs-delivered antigens are useful in antigen cross-presentation to cytotoxic T lymphocytes (CTLs) and development of robust cell-mediated immune response [58, 59]. PLGA-based particulate vaccines are shown to induce efficient T-cell immunity in mice and pigs [60–63]. Similarly, rodent and pig studies have shown that polyanhydride NPs-based vaccines also enhance cellular immunity [50, 64]. Thus, immunogenic properties of different polymer-based NPs could be exploited to improve the efficacy of vaccines for use against porcine viral infections.

## Particulate vaccines and porcine viral infections

In this review, only studies conducted in pigs related to the development and evaluation of NPs-based vaccine candidates by using virus-like particles (VLPs), biodegradable polymers, polysaccharides and liposomes against porcine viral infections are included (Table 3).

**Table 3:** **NPs-based vaccines tested against different porcine viruses**

Target virus	Antigens	NPs	Adjuvant	Route	Results	References
Porcine reproductive and respiratory virus (PRRSV)	GP5, GP4, GP3, GP2a and M proteins	VLPs	*M. tuberculosis* WCL	IN	Enhanced IFNγ response; partial protection	[70]
	N, M, GP5 and E proteins	VLPs	*2′,3′*-*cGAMP VacciGrade™* (Invivogen, USA)	IN	Enhanced viremia associated with IFNα	[71]
	Whole inactivated virus	PLGA	None	IN	Increased number of NK cells, γδ cells and IFNα cytokine; enhanced CD8^+^ T cells and IFNγ response; better protection against homologous and heterologous challenge	[86, 88]
	Whole inactivated virus	PLGA	*M. tuberculosis* WCL	IN	Robust cell-mediated and antibody responses; improved heterologous protection	[87, 89]
Swine influenza A virus	HA, NA, M1	VLPs	*Emulsigen* (MVP Lab, USA)	IN	Induction of serum IgG, mucosal IgA and virus neutralizing antibody responses; protection from homologous challenge	[72]
	Conserved peptides	PLGA	*M. vaccae* WCL	IN	Enhanced epitope-specific T cell response; reduced virus load in lungs	[61]
	Whole inactivated virus	PLGA	None	IN	Enhanced CD8^+^ T cells and IFNγ production; reduced virus load in lungs	[62]
	Whole inactivated virus	Chitosan	None	IN	Enhanced mucosal IgA and cellular immunity; reduced virus load in lungs and also reduced virus shedding from nostrils	[105]
	Whole inactivated virus	Nano-11	None	IN	Enhanced cross-reactive mucosal IgA antibody response	[108]
	Whole inactivated virus	Polyanhydride	None	IN	Cellular response better than inactivated virus vaccine	[64]
	Whole inactivated virus	Polyanhydride	CpG-ODN	IN	Enhanced mucosal cell-mediated and IgA antibody responses and reduced virus titers from respiratory tract after heterologous challenge	[95]
	Conserved 10 peptides	Liposome	Monosodium urate crystals	IN	Improved mucosal IgA and cell-mediated immune responses, better protection than soluble peptides alone	[113]
	Whole inactivated virus	Nano-11	None	IN	Induced cross-reactive mucosal IgA antibody response	[108]
Porcine epidemic diarrhea virus (PEDV)	Whole inactivated virus	PLGA	None	IN	Cellular and humoral immunity and protection better than inactivated vaccine with adjuvant	[90]
Foot and mouth disease virus (FMDV)	T-cell epitope of 3A protein	VLPs	*Montanide ISA 206* (Seppic)	IM/IN	Induction of better serum IgG and IgA antibody responses and increased number of IFNγ secreting cells and cell proliferation	[73]
	Capsid proteins VP0, VP1, VP3	VL Ps	*Montanide ISA 206* (Seppic)	IM	Enhanced neutralizing antibody production and IFNγ response in PBMCs, complete homologous protection	[74]
	VP1 peptides (EP141-160)	VLPs	Freund’s adjuvant	IM	Virus-specific neutralizing antibody production and protection (60%) lower than inactivated vaccine (80%)	[75]
	VP1 epitopes	VLPs	None	IM	Induction of higher neutralizing antibody production and better protection compared to synthetic peptides group	[76]
Encephalomyocarditis Virus (EMCV)	P1, 2A and 3C proteins	VLPs	*Montanide IMS 1313* *N VC* (Seppic)	IM	Antibody response comparable to commercial vaccine	[21]
Japanese encephalitis virus (JEV)	prM and E proteins	VLPs	*ISA 201* (Seppic)	SQ	Induction of neutralizing antibody response and protection from homologous (genotype I) and heterologous (genotype III) challenge virus infection	[22]
Porcine circovirus 2 (PCV2)	Cap protein	VLPs	*Montanide ISA 201* (Seppic)	IM	Induction of cap-specific IgG antibodies; protected from clinical signs of disease	[77]
	Cap protein and somatostatin	VLPs	None	SQ	Induction of cap-specific IgG antibody; reduced clinical signs and viremia	[78]
	Cap protein and GM-CSF	VLPs	*Montanide ISA206* (Seppic)	IM	Enhanced neutralizing antibody production and improved weight gain after challenge infection compared to control	[79]
Porcine parvo virus (PPV)	VP2 protein	VLPs	Mineral oil	IM	Reduced virus transmission from sows to fetuses and induced neutralizing antibody production in weaned piglets	[80]

IN: intranasal, IM: intramuscular, SQ: subcutaneous.

### Virus-like particles (VLPs)

VLPs are constructed using viral structural proteins, which can self-assemble but are non-infectious as they lack the viral genomic material. VLPs mimic the virion and can effectively induce innate and adaptive immune responses [65]. VLPs are produced using different bacterial, insect, yeast or mammalian expression systems [66]. Due to their smaller size and particulate nature, VLPs-based vaccines are processed and presented not only through MHC class II but also through MHC class I pathway leading to the generation of antibodies as well as CTL responses [67, 68]. The potential use of VLPs in porcine viral vaccine development is evident through the success in commercialization of Human Papilloma Virus (HPV), Hepatitis B virus and malaria vaccines by adapting this technology [69].

In one study, PRRSV VLPs containing five (GP5, GP4, GP3, GP2a and M) and two (GP5 and M) viral surface proteins were generated using the baculovirus expression system. PRRSV VLPs vaccine was mixed at 1:1 ratio with *Mycobacterium tuberculosis* whole cell lysate (*M. tuberculosis* WCL) adjuvant and administered INto pigs. VLPs-vaccinated pigs were partially protected with 2-log reduction of virus titers in lungs. VLPs-vaccinated pigs also had enhanced IFN-γ response compared to mock challenge pigs [70]. However, in another study, when pigs were vaccinated IN with PRRSV VLPs expressing N, M, GP5 and E proteins, enhanced viremia accompanied with higher level of IFN-α cytokine response was observed [71]. The contrasting results in PRRSV VLPs study suggest the need for further research to fully evaluate the potential of VLPs-based PRRSV vaccines for swine.

Influenza-associated VLPs expressing HA, NA and M1 proteins of pandemic 2009 (H1N1) virus were inoculated twice intramuscularly with or without *Emulsigen* (MVP Lab, USA) adjuvant to pigs. This vaccine induced robust serum IgG, mucosal IgA and virus neutralizing antibody responses in pigs. After homologous virus challenge, VLPs-vaccinated pigs had significantly reduced pneumonic lesions and virus titers were substantially lowered in upper and lower respiratory tracts compared to mock vaccinated animals [72].

Many studies have been conducted with the goal to develop VLPs-based FMDV vaccine using various expression systems encoding different viral antigens. Rabbit hemorrhagic disease virus (RHDV) VLPs expressing T-cell epitope of 3A protein of FMDV (RHDV-3A-VLPs) was generated. This VLPs vaccine induced maturation of bone marrow derived dendritic cells in vitro [73]. Pigs immunized IM with RHDV-3A-VLPs together with *Montanide ISA 206* adjuvant (Seppic, France) induced higher serum IgG and IgA antibody responses. This vaccine also increased number of IFN-γ secreting cells and lymphoproliferative responses in PBMCs compared to vaccine delivered without adjuvant and IN RHDV-3A-VLPs inoculated pigs; however, challenge experiments were not performed [73]. Guo et al. constructed FMDV VLPs expressing capsid proteins VP0, VP1 and VP3 and immunized pigs by IM route [74]. VLPs-vaccinated pigs produced virus-specific neutralizing antibodies and IFN-γ response in peripheral blood mononuclear cells (PBMCs) as good as the inactivated FMDV vaccine control. After challenge with homologous virus, VLPs-vaccinated pigs did not show specific clinical signs [74]. In another study, VP1 epitope peptides (EP141-160) of FMDV were inserted into the coat protein genes of male-specific coliphage (MS2) (CP-EP141-160 VLPs) and injected IM to pigs. This formulation resulted in induction of virus neutralizing antibodies and protected 60% of the immunized pigs compared to only 20% protection in peptide alone vaccinated animals. However, the protection was lower than inactivated vaccine (80%) indicating the need of further improvement in this VLPs either by using longer sequence of epitope or addition of other adjuvants [75]. VLPs generated by insertion of VP1 epitopes of FMDV into porcine parvovirus VP2 were administered IM to pigs. This VLPs-vaccine induced higher virus neutralizing antibodies compared to synthetic peptide vaccine and resulted in better protection to challenge FMDV infection [76].

VLPs have also been developed and tested against porcine neurotropic viruses [21, 22]. Porcine encephalomyocarditis virus (EMCV) VLPs containing structural protein P1, nonstructural protein 2A and protease 3C were generated. After IM administration together with *Montanide IMS 1313* *N VG* adjuvant (Seppic), VLPs-vaccine induced sustained production of virus neutralizing antibodies comparable to commercial vaccine control. There was absence of any severe injection site reactions in VLPs-vaccinated pigs [21]. This suggests the potential of developing VLPs-based vaccine against EMCV disease in pigs. Likewise, in a recent study, Japanese encephalitis virus genotype I (GI) VLPs encoding premembrane (prM) and envelope (E) proteins were constructed. After subcutaneous immunization, this vaccine formulation induced robust neutralizing antibody response and protection against both homologous GI and heterologous GIII JEVs viruses. This finding indicates the cross-protection potential of VLPs-based JEV vaccine in pigs [22].

Early study on PCV2 VLPs used full length Cap protein in *Escherichia coli* expression system [77]. Pigs vaccinated against PCV2 using Cap VLPs and *ISA 201* adjuvant (Seppic) by IM route induced Cap-specific IgG antibodies. Vaccinated animals were apparently healthy with normal body weight gain and absence of any clinical signs of disease [77]. Li et al. [78] showed induction of Cap-specific IgG antibodies in pigs vaccinated by subcutaneous (SC) injection of Cap VLPs. Vaccinated pigs demonstrated reduced fever, viremia and mild pathological changes in lungs and lymph nodes compared to unvaccinated challenge animals. In another study, VLPs co-expressed with Cap protein and porcine GM-CSF were administered IM to pigs. This vaccine formulation induced significantly higher virus neutralizing antibodies in pigs. After virus challenge, VLPs-vaccinated pigs had normal body weight gain compared to Cap protein alone and commercial PCV2 vaccine groups. Virus clearance, however, was observed in equally in VLPs as well as other control vaccine groups [79].

Only a single VLPs-based vaccine study for porcine parvovirus was found [80]. PPV-VLPs expressing major structural protein VP2 were administered IM with double oil emulsion (DOE) mineral oil adjuvant to weaned pigs. There was an induction of significantly higher neutralizing antibodies in VLPs-vaccinated animals compared to inactivated vaccine group. Further, when gilts immunized with this formulation were challenged with virulent PPV, virus was not detected in any of the fetuses. Thus, PPV-VLPs can be a potential vaccine candidate to prevent PPV-induced reproductive failure [80]. In summary, VLPs of various origin can be used to develop more efficient vaccines against porcine viral infections. Further studies are needed to evaluate their immunogenicity and protective efficacy under field conditions.

### Biodegradable synthetic PLGA NPs

PLGA is a co-polymer of lactic acid and glycolic acid. It is the most widely explored synthetic polymer in vaccine studies. It is a safe and non-toxic compound, and its hydrolysis products are readily assimilated into existing metabolic pathways [81]. PLGA nanoparticles are prepared either by oil in water emulsification or nanoprecipitation methods [82, 83]. PLGA NPs bear a net negative charge. They enter APCs through pinocytosis and endocytosis, undergo reversal of charge and endo-lysosomal escape of entrapped vaccine cargo leading to antigen processing in cytoplasm, resulting in cross-presentation of antigen to CD8^+^ T cells through MHC class I pathway [59, 82]. PLGA NPs are involved in maturation of DCs of mice and human origin, and controlled release of entrapped antigens leading to efficient expansion and differentiation of memory T-cells [84, 85]. In rodent studies, induction of robust T-cell immunity is observed with PLGA NPs-based vaccines containing various vaccine antigens [55, 82]. Further, PLGA is approved for drug deliveries in humans by the US Food and Drug Administration (FDA) and European Medicine Agency (EMA) [82].

PLGA NPs enhance antigen uptake and induce maturation of porcine APCs [62, 86, 87]. Single dose of IN immunization with PLGA NPs-encapsulated inactivated/killed PRRSV antigen (NPs-KAg) induced activation of innate natural killer (NK) cells, γδ T-cells and secretion of innate cytokine IFNα [86]. NPs-KAg vaccine also induced greater frequency of CD8^+^ T cells; increased secretion of IFN-γ; lowered frequency of T-regulatory cells; and reduced secretion of inflammatory cytokines compared to control KAg-vaccinated animals [86, 88]. In a subsequent study, when NPs-KAg was co-administered IN with *M. tuberculosis* WCL adjuvant, a balanced Th1/Th2 immune response and augmentation of mucosal IgA antibody response was observed. After heterologous PRRSV challenge, pigs that received NPs-based vaccine showed no clinical signs and also had significant reduction in lung virus load [87, 89].

PLGA NPs were also used to encapsulate highly conserved influenza peptides and evaluated for efficacy in pigs after IN administration. PLGA NPs-based subunit vaccine resulted in induction of epitope-specific T-cell response but not the antibody response [61]. The T-cell biased immune response was also observed in pigs after IN immunization with PLGA NPs-encapsulated inactivated/killed influenza virus (PLGA-KAg) vaccine in pigs [62]. In PLGA-KAg vaccine administered animals observed reduced fever; lowered pneumonic lesions; and increased virus clearance from lungs after heterologous virus challenge compared to KAg vaccine controls [62].

In another study, PEDV KAg was encapsulated in PLGA NPs and used to immunize pregnant sows by IN route. This nanovaccine induced higher virus-specific IgG and neutralizing antibodies in serum and greater IgG, IgA and neutralizing antibody responses in colostrum. It also induced greater cell proliferation and IFN-γ responses in restimulated PBMCs compared to KAg vaccine controls. Importantly, piglets born to NPs-vaccinated sows had higher virus neutralizing antibodies and were better protected against homologous virus challenge than KAg controls [90]. These studies suggest that PLGA NPs can be used as an efficient means of enhancing virus-specific cell-mediated immune responses in pigs.

### Polyanhydride-based NPs

Polyanhydrides are another type of synthetic polymer widely studied for vaccine deliveries [91]. Polyanhydride NPs are synthesized by polycondensation or emulsification processes and are biodegradable, biocompatible and safe for vaccine delivery [91, 92]. They activate innate immune responses in a manner similar to lipopolysaccharides (LPS) [93]. The surface-eroding nature of polyanhydride NPs provides safe microenvironment for the encapsulated antigens and facilitates slow and sustained antigen release [92, 94]. Induction of better antibody and cell-mediated immune responses by polyanhydride NPs-based vaccines has been reported against viral, bacterial and parasitic infections [48, 91]. Inoculation of polyanhydride NPs-based SIV KAg vaccine (KAg-nanovaccine) by IN route enhanced cell-mediated but not the antibody responses in pigs [64]. After heterologous virus challenge, KAg-nanovaccine group had six to eightfold reduction of nasal virus shedding compared to KAg vaccine controls [64]. In a subsequent study, when KAg-nanovaccine formulation was supplemented with CpG-ODN adjuvant, both cell-mediated as well as mucosal IgA antibody responses were improved [95]. After heterologous virus challenge, CpG-ODN-adjuvanted KAg-nanovaccine provided better protection through a significant reduction in influenza-induced fever, 16-fold reduction of nasal virus shedding and 80-fold reduction in lung virus titers compared to pigs immunized with five-times greater quantity of soluble killed antigen (KAg) vaccine [95]. This study also indicates the dose-sparing ability of polyanhydride NPs. Thus, polyanhydride NPs can also be used to induce better cellular as well as humoral immune responses in pigs.

### Polysaccharide-based NPs

Chitosan, alginate and other polysaccharides have also attracted attention as materials for NPs formulation and drug delivery studies. Chitosan is a natural polymer derived from deacetylation of chitin and is composed of glucosamine and *N*-acetylglucosamine residues [96]. Due to the availability of amino and carboxyl groups in an acidic microenvironment, chitosan NPs have net positive surface charge which makes them highly mucoadhesive and increases their half-time of antigen retention on mucosal surfaces [97, 98]. Further, chitosan NPs can reversibly open the epithelial cell tight junctions thereby improving paracellular and intracellular antigen transport across mucosal epithelial surfaces [99, 100]. Chitosan NPs also enhance antigen uptake by APCs, induce APC maturation and active secretion of innate cytokines [101, 102]. Thus, chitosan NPs form an attractive mucosal vaccine delivery vehicle.

Chitosan-based NPs are used in pigs to deliver adjuvants such as bee venom and plasmid encoding porcine IL-2 and IL-4/IL-6 genes, which improved induction of better virus-specific immune responses of respective vaccines against PRRSV and PCV2 [103, 104]. Chitosan NPs enhance antigen uptake by porcine APCs and activate them to produce innate cytokines including IFN-alpha, TNF-alpha and IL-1β [105]. Chitosan NPs encapsulated SIV KAg (CNPs-KAg) vaccine administered twice through IN route without any additional adjuvant in pigs induced the cross-reactive mucosal IgA antibodies. Chitosan NPs-based vaccine also induced IFN-γ response in PBMCs and tracheobronchial lymph nodes (TBLN) better than KAg vaccine controls. This vaccine formulation substantially reduced the challenge heterologous virus titers by up to 100-fold in both the upper and lower respiratory tracts compared to soluble KAg vaccine. This finding emphasizes the potential benefits of using Chitosan NPs in future development of mucosal swine influenza vaccine for pigs [105].

Recently, dendrimer-like-alpha-d-glucan (Nano-11) NPs derived from sweet corn variety *sugary*-*1* was examined as an alternative, safe, cost-effective and potent adjuvant [106, 107]. Nano-11 are positively charged NPs which efficiently adsorb negatively charged antigens through electrostatic interactions. Rodent studies have shown that Nano-11 NPs enhance antigen uptake by DCs, induce their maturation, activate them to produce pro-inflammatory cytokines and help in induction of antigen-specific antibodies [106, 107]. In a recent study, we observed that Nano-11 NPs with or without addition of SIV killed antigen (KAg) can stimulate porcine APCs and produce cytokines such as IFN-α, TNF-α and IL-1β [108]. Pigs immunized via IN route with Nano-11 NPs adsorbed SIV KAg at two-to-one ratio (Nano-11 + KAg) resulted in cross-reactive mucosal IgA responses better than KAg controls. Moreover, pigs immunized IM with Nano-11 adsorbed ovalbumin (Nano-11 + OVA) had significantly greater IgG1 and IgG2 antibodies in serum compared with pigs vaccinated with OVA alone [108]. These findings highlight the possibility of using corn-derived Nano-11 NPs as a potential adjuvant in porcine viral vaccine development.

### Liposome-based NPs

Liposomes can encapsulate both hydrophilic and hydrophobic molecules in aqueous and non-aqueous phases of their vesicles [109]. Liposome vesicles protect antigens from enzymatic degradation, enhance antigen internalization by APCs and maintain controlled release of antigens [110]. Liposome-encapsulated antigens can enhance both cellular and humoral immune responses [110, 111]. In a pig study, liposome NPs were used as an IM adjuvant for a PCV2 DNA vaccine [112]. Liposome NPs-adjuvant induced higher neutralizing antibodies and IFNγ response in pigs and reduced viremia of a challenge virus compared to alum-adjuvanted vaccine, providing the evidence that liposome NPs can be a potent adjuvant in pigs [112]. In our recent study, we used liposome NPs to encapsulate ten highly conserved peptides of different influenza viruses of human and pig origin and immunized pigs through IN route co-administered with monosodium urate (MSU) crystal adjuvant [113]. The liposome-adjuvant based vaccine enhanced the mucosal IgA antibody response and induced peptide and virus-specific T-helper/memory cells and IFNγ responses resulting in reduced fever and modest reduction in virus titers in the respiratory tract of pigs [113]. These studies highlight the fact that liposome-based NPs can be used as an attractive vaccine delivery platform against porcine viral infections.

## Conclusions and future perspectives

Virus infections have significant impact on pig industry worldwide. Use of available vaccines have definitely helped in achieving strong control over some of the porcine viral infections such as Food and Mouth Disease, Transmissible Gastroenteritis, Classical Swine Fever and Pseudorabies. Vaccination also helped in reducing the clinical signs and increasing the production parameters in PCV2-associated disease. However, for many other porcine viruses, further improvements in existing vaccine platforms and development of novel vaccine delivery systems are necessary to: (1) Induce better mucosal and cell-mediated immunity; (2) Protect against emerging and re-emerging strains; (3) Enhance the breadth (heterologous, cross-genotype and heterosubtypic) of immunity; and (4) Differentiate between infected and vaccinated animals.

NPs-based vaccine delivery platforms such as VLPs, biodegradable polymers and liposomes have great potential as they—(1) Protect vaccine antigens from degradation; (2) Facilitate antigen uptake and processing by APCs; (3) Impart adjuvant potential; (4) Can be used in mucosal and other alternate routes of immunizations; and (5) Induce effective mucosal and cellular cross-protective (broader) immunity. Research efforts are ongoing to develop porcine viral vaccines using NPs-based technologies. However, more collaboration(s) and in-depth studies are warranted to make this innovative vaccine antigen delivery technology successful and practical for application in food animal industry. To date, almost all of the immunomodulatory mechanisms of NPs-based vaccine delivery platforms have been studied in rodent disease models, which may or may not reflect the situation in pigs or other domestic animal species [114]. Likewise, proper understanding of effect of size, charge and other physicochemical properties of NPs after delivery through different routes of immunization in pigs is necessary to make efficient translation of this robust NPs-based vaccine technology. Similarly, studies should also focus on NPs stability at different storage conditions and immunogenicity over a long period of time as they will directly associate with commercial aspect of the vaccine product. Recent advances in NPs-based adjuvant and vaccine delivery platforms in pigs demonstrate great promise to yield better candidate vaccines against many porcine viral infections with enhanced efficacy in the field. These nanovaccine technologies can also be adopted to develop effective vaccines against viral infections in other animal species, and knowledge gained could be exploited for improving the efficacy of existing human viral vaccines.
